# ‘PULSE FICTION’: Development of Slightly Processed Pulse‐Based Foods and Recipes to Meet the Needs of Consumers and the Agricultural Sector and Improve Food Sustainability

**DOI:** 10.1111/nbu.70041

**Published:** 2026-01-07

**Authors:** Gaëlle Arvisenet, Stéphanie Chambaron, Zaira Hernandez‐Casiano, Hélène Gerard‐Simonin, Corinne Tanguy, Clémentine Hugol‐Gential, Anne Saint‐Eve, Christian Salles

**Affiliations:** ^1^ Université Bourgogne Europe Institut Agro, CNRS, INRAE, UMR CSGA Dijon France; ^2^ UMR PAM, L'institut Agro, INRAE Université Bourgogne Europe Dijon France; ^3^ UMR CESAER, INRAE, L'institut Agro Université Bourgogne Europe Dijon France; ^4^ CIMEOS, Maison Des Sciences de L'homme de Dijon Université Bourgogne Europe Dijon France; ^5^ Université Paris‐Saclay, AgroParisTech, INRAE UMR SayFood Palaiseau France

**Keywords:** consumers, culinary applications, environmental impact, farmers, intermediate food products (IFPs), minimally processed foods, pulses

## Abstract

Pulses offer significant nutritional and environmental benefits and are useful components of healthier, more sustainable diets and global food security. However, their consumption in France remains low and below the world average. Farmers face economic and technical challenges in diversifying crops, and current domestic production is insufficient to meet this low demand, resulting in continued reliance on imports. Production and consumption are closely linked: low consumer demands limit incentives for farmers to expand cultivation, while limited availability can also constrain uptake. One of the major barriers to consumption relates to preparation and processing. While consumers generally view raw or minimally processed pulses positively, they often avoid using them because they perceive them as time‐consuming to prepare or associate them with undesirable sensory attributes (e.g., bitterness and astringency). It also might be difficult for consumers to categorize pulses according to their level of processing. Although pulse processing makes them more convenient for users, former studies suggest that consumers are suspicious about the processing of pulses, and that they may even confuse processing with ultra‐processing. This suspicion could sometimes lead to rejection by consumers. The process should thus remain moderate. Encouraging the use of minimally processed pulses, which balance convenience and acceptance, is crucial for wider adoption. The aim of the PULSE FICTION project is to develop minimally processed pulse products with pulses as the main ingredient that align with consumer preferences, farmer constraints and sustainability goals. A key innovation will be the selection of intermediate food products (IFPs) on the basis of consumer and farmer input and chef‐developed easy‐to‐make recipes. These products will be evaluated for their nutritional and sensory qualities, satiety, consumer acceptance and environmental impact. Beyond product development, PULSE FICTION explores the cognitive and sensory factors influencing consumer acceptability and designs effective communication strategies for all stakeholders to facilitate consumer adoption of pulse‐based foods.

## 1 Introduction

Pulses are very important for achieving a healthier and more sustainable diet by reducing the reliance on animal‐based foods, enhancing food security and improving sustainability (Foyer et al. 2016). Including pulses in cropping systems and increasing their quantity in European diets is beneficial for the environment and health (Poore and Nemecek 2018; Nemecek et al. 2008; Reckling et al. 2016), making them central in dietary forecasting models, particularly in sustainable diet scenarios such as those proposed by the EAT–Lancet Commission (Willett et al. 2019). However, pulse consumption in France remains low, only 2 kg per person annually (2020–21), which is well below the global average of 7.6 kg per person (Rawal and Navarro 2019; Agreste 2022). Paradoxically, French production does not even meet this low demand, and most pulses used for human consumption need to be imported. Indeed, protein crop cultivation represents < 3% of agricultural land in France and Europe. France produces 1 million tonnes of protein crops (peas, faba beans, soybeans) annually, making it the leading producer and exporter of peas and faba beans in Europe. However, France remains highly dependent on imports because it only produces half of the plant‐protein‐rich materials needed to feed its animals (soybean, rapeseed, sunflower meal, etc.) and only a third of those intended for human consumption (Comité d'évaluation du Plan France Relance 2024). Expanding pulse cultivation in France could benefit the environment (soil, water, CO_2_ emissions and biodiversity) (Guinet et al. 2020; Voisin et al. 2014) and create new market opportunities for the food industry with more outlets.

The process of crop diversification in favour of pulses faces technical and economic challenges that make the conversion of pulse cultivation risky for farmers because of limited technical advice and uncertain financial returns. Pulse cultivation may become profitable only if the consumption of pulses and pulse‐based products increases in the French population. This lack of incentives may hinder the adoption of pulse production. Moreover, new agronomic methods must be implemented, and new crop varieties need to be found to resist pest attacks and adapt to climate change. The acceptance of these new varieties by consumers who are unfamiliar with them is another matter of uncertainty. Encouraging production and increasing consumption are thus intrinsically interconnected issues because the quantity of pulses to be produced depends on assumptions about the evolution of the French diet (Magrini et al. 2018), and the availability of locally produced pulses may encourage greater adoption of these products by consumers.

Therefore, it appears crucial to increase the consumption of pulses to favourably impact the national production of these crops and, in turn, provide more incentives to French farmers to grow them by ensuring outlets for pulses through their more frequent and regular consumption. Our point of view is that efforts that address pulse production and diversification will not be successful if ways to increase the human consumption of pulses are not sought in parallel. A better understanding of the obstacles to pulse consumption among French consumers is thus needed to implement support strategies.

The following sections describe the rationale and structure of the PULSE FICTION project, organised into six complementary work packages. Each work package addresses a specific facet of the challenge—from understanding consumer barriers and farmer constraints to product development, sustainability assessment and consumer communication—in order to propose a holistic, actionable approach to increasing pulse consumption and production in France.

### 1.1 Understanding Consumer Barriers to Pulse Consumption

Efforts to increase pulse production will be ineffective unless consumption increases simultaneously. Prior research (Melendrez‐Ruiz, Buatois, et al. 2019; Melendrez‐Ruiz, Chambaron, et al. 2019; Melendrez‐Ruiz et al. 2020, 2022; Melendrez‐Ruiz, Claret, et al. 2021; Melendrez‐Ruiz, Goisbault, et al. 2021) highlights that consumer attitudes and perceptions of pulse processing remain major barriers (Melendrez‐Ruiz et al. 2022). Paradoxically, even though they express positive attitudes towards raw or minimally processed pulses, nonvegetarian French consumers rarely consume these products because of perceived difficulties in preparation (Melendrez‐Ruiz, Buatois, et al. 2019). Moreover, unprocessed pulses are sometimes rejected for their sensory attributes, such as green notes, bitterness and astringency (Karolkowski et al. 2021, 2023). In addition to individual consumer barriers, pulses also remain underrepresented in institutional food services. Recent research has shown that in France, pulses are rarely served in collective catering and that cooking and sourcing practices do not yet support their integration into sustainable diets (Magrini et al. 2021). This structural underuse reduces exposure to pulses in everyday meals and limits the development of familiarity and culinary skills among consumers.

To improve convenience, the food industry has developed processed pulse products, such as lentil‐based ham alternatives, chickpea chips or bean spreads (Métayer and Denhartigh 2016). Moreover, researchers have explored plant protein extraction for use as food ingredients, emulsifiers, stabilisers and fortificants (Matser et al. 2023). These refined ingredients also increase protein digestibility and reduce undesirable flavours (De Angelis et al. 2020). However, ultra‐processing might not be the best answer to consumers' expectations. Ultra‐processed foods (UPFs) are defined by O'Connor et al. (2024) as ‘industrially manufactured products made up of several ingredients (formulations) including sugar, oils, fat and salt (generally in combination and in higher amounts than in processed foods) and food substances of no or rare culinary use’. First, products containing plant protein powders are not well understood by consumers, who do not spontaneously associate the concept of plant proteins with pulses (Melendrez‐Ruiz et al. 2020). Moreover, the increased intake of ultra‐processed foods has been linked to cardiovascular disease (Srour et al. 2019a, 2019b), cancer (Fiolet et al. 2018) and increased mortality (Schnabel et al. 2019). Despite the convenience of processed pulses, negative perceptions of ultra‐processed pulse‐based products persist (Melendrez‐Ruiz et al. 2022; Ares et al. 2016; Varela et al. 2022; Chollet et al. 2022).

Moreover, the refining processes that are needed to produce plant proteins as ingredients for processed foods are much less sustainable than the direct consumption of pulses. Indeed, primary processing of protein isolates requires solubilisation, extraction and drying; all these processes use a significant amount of added water, which is followed by a particularly high‐energy‐consuming drying step. The isolation of protein isolates also wastes material and is expensive (Matser et al. 2023).

### 1.2 The Need for a New Approach

Consequently, the increase in plant proteins in the human diet should not be solely based on the addition of proteins extracted from pulses that are used as ingredients in ultra‐processed products. The consumption of pulses as a food product should be encouraged. To achieve this, it is necessary to reconcile practicality and acceptable organoleptic quality without processing the product too much. Slight processing, which makes pulses both easy to use and acceptable to consumers, seems to be the cornerstone for improving the acceptance and adoption of pulses. Surprisingly, finding new pulse processes that could be better accepted by consumers has received little attention from researchers, despite their potentially strong consequences for dietary behaviors. Solutions based on the culinary appeal of pulse‐based products, simple preparation at home with common kitchen utensils and in less than an hour, and communication focused on their gustatory and organoleptic assets would certainly be a complementary way to enable consumers to (re)discover these unfairly neglected products, outside of the typical ultra‐processed formats that some consumers find confusing or unappealing.

### 1.3 PULSE FICTION: A Collaborative Approach to Pulse Innovation

**FIGURE 1:**
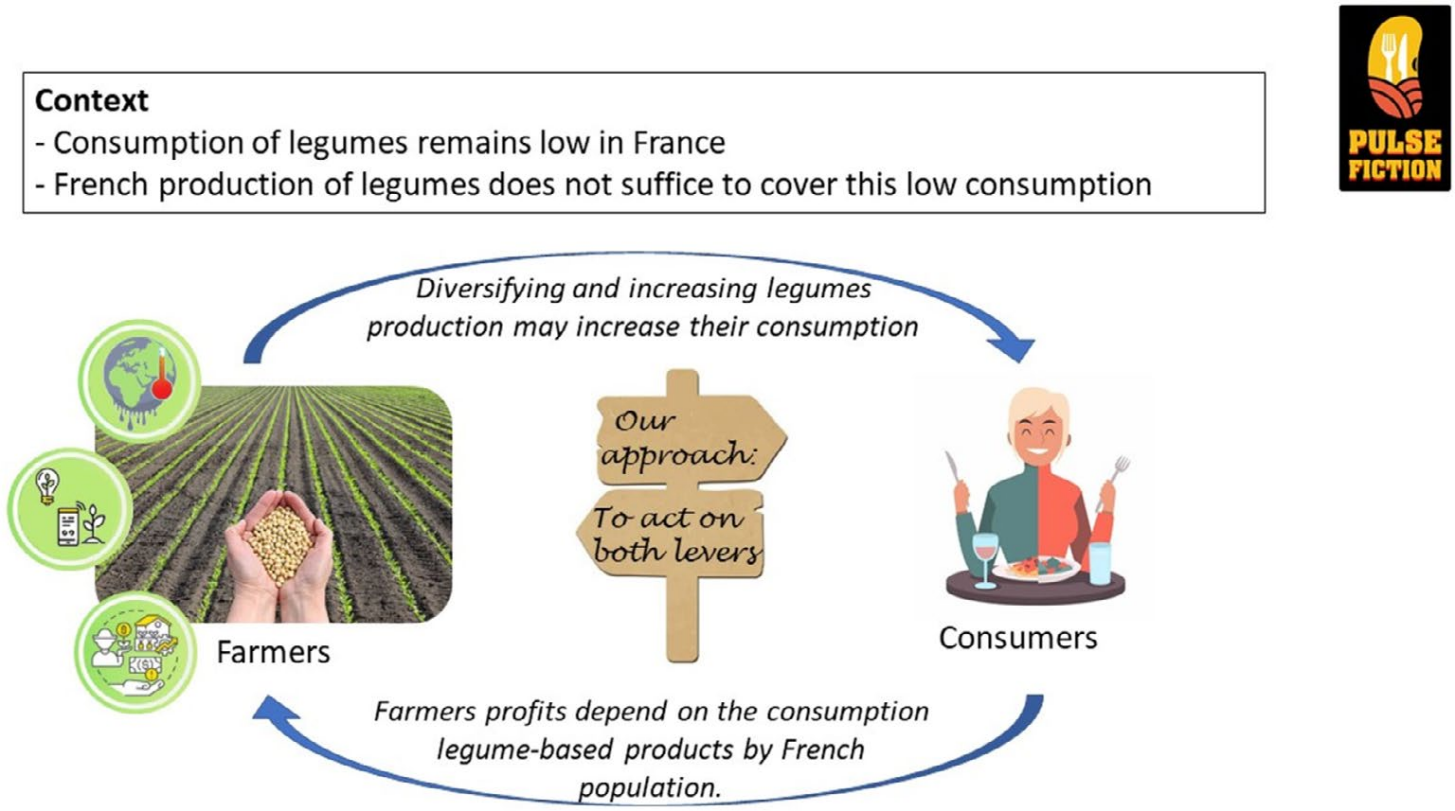
Original approach of PULSE FICTION: Acting at both the consumer and farmer levels.

**FIGURE 2:**
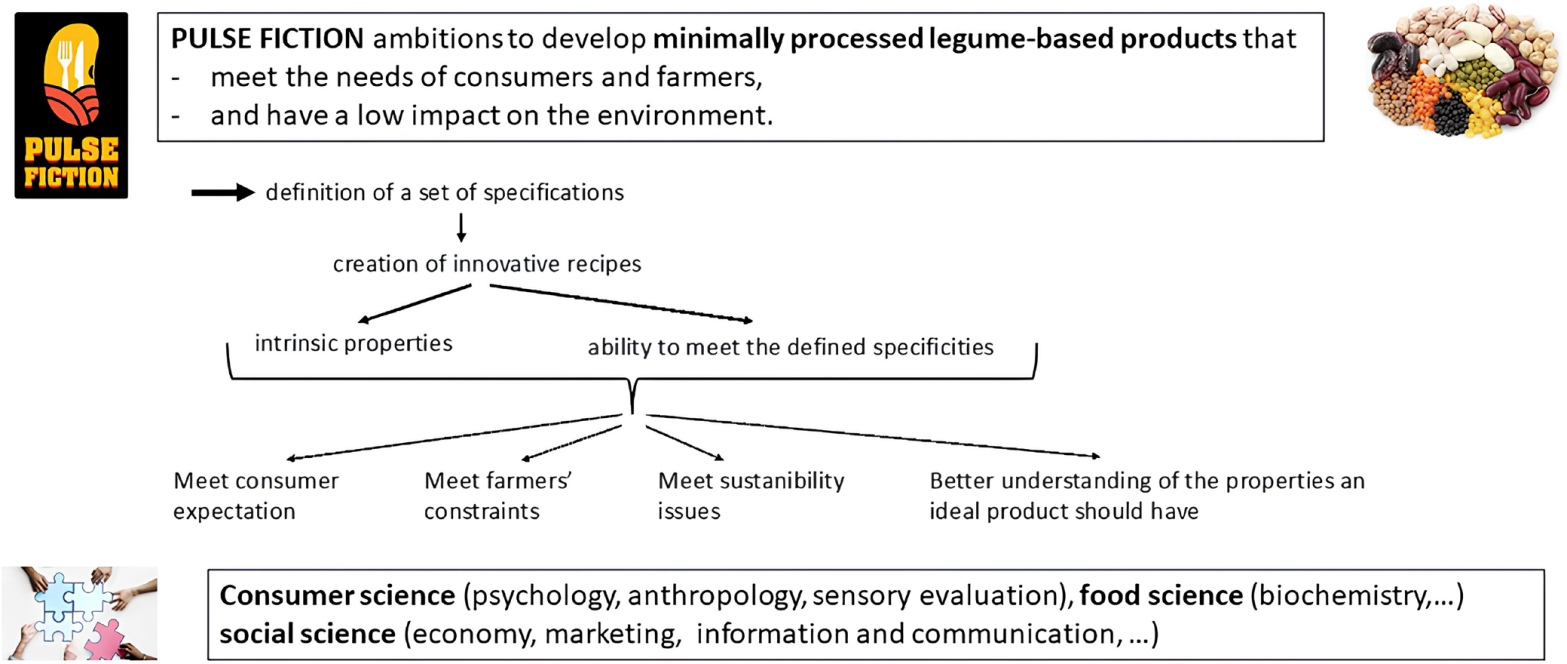
General objective of PULSE FICTION.

PULSE FICTION aims to develop minimally processed pulse‐based products that align with consumer preferences, farmer constraints and environmental sustainability (Figure 1). These pillars will guide the creation of innovative recipes that are analysed for their nutritional, sensory and environmental properties. This multidisciplinary initiative integrates consumer sciences (psychology, anthropology and sensory evaluation), food science (biochemistry) and environmental and social sciences (economics, communication and behavioural studies) (Figure 2). This integrative approach echoes the emerging concept of ‘alimentation’, which advocates for a unified understanding of food‐related phenomena across biological, technological and socio‐cultural domains (Aguilera 2021).

The main objectives are as follows:

– Identify consumer expectations regarding acceptable levels of processing for pulse‐based products.
– Explore the constraints faced by farmers in carrying out a prospective analysis of their future needs and the foreseeable evolution of the sector.
– Adapt the multi‐criteria analysis of territorialisSed food chains to the specificities of pulse‐based products.
– Create and characterise minimally processed products with low environmental impacts that are adapted to consumers' and farmers' expectations and constraints.
– Identify key acceptability markers by considering cognitive, sensory, financial, and digestive factors.
– Develop consumer engagement strategies to encourage home preparation of minimally processed pulses.

**FIGURE 3:**
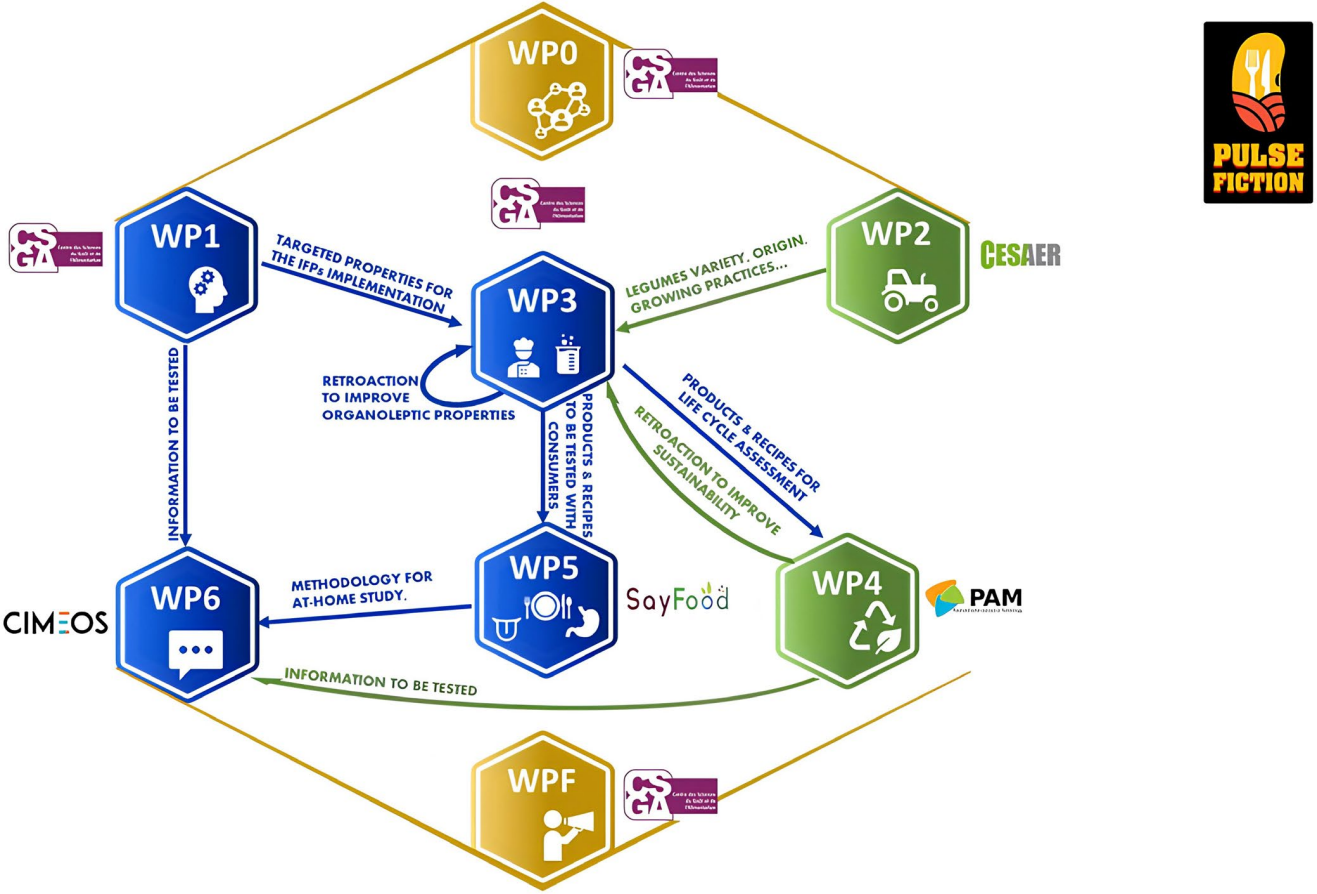
Global organisation of PULSE FICTION. WP0 is for the management of PULSE FICTION; WPF is for dissemination and communication activities.

The main structure of PULSE FICTION is presented in Figure 3 and a summary of Pulse Fiction organisation, including the main expected outcomes, is presented in Table 1.

**TABLE 1:** Summary of Pulse Fiction organisation: Work Packages, key tasks and expected outcomes.

Work package (WP)	Key tasks	Expected outcomes/goals
WP1	T1.1—Identification of mental representations and attitudes towards legumes with different levels and types of processing. T1.2—Observation of current consumers' culinary practices concerning home‐preparation of legumes. T1.3—Identification of consumer‐centred solutions and cocreation for WP3.	Study of mental representations and attitudes of French consumers towards pulse‐based food products that have undergone various levels of processing. Study of the cultural practices and drivers of pulse consumption, from purchase to consumption, focusing on knowledge, know‐how and associated techniques. Provide guidelines for the implementation of WP3 recipes. Contribution to the selection of types of pulses to be studied in WP3, based on consumer expectations and preferences.
WP2	T2.1—Identification of the main actors, territories and characteristics of legume production and processing in France and Bourgogne Franche‐Comté (BFC). T2.2—Determination of the main barriers related to the production and processing of legumes and identification of innovations and initiatives in agri‐food chains. T2.3—Collection data to contribute to life cycle assessment (WP4).	Study on evolution of each pulse identified: productions, cultivated areas and yields in France and BFC and determine the production potential of pulses destined for human consumption in Bourgogne‐Franche‐Comté or nearby. Recommend the most locally adapted pulse varieties in BFC for human consumption (to be used in WP3). Identify the current and future key players in the production and processing of pulses in the region and their location. Contribution to scenarios of territorialized pulse‐based food chains in BFC for WP4.
WP3	T3.1—Selection and processing of pulses into intermediate food products (IFPs). T3.2—Characterisation of the IFPs. T3.3—Integration of the IFPs in culinary recipes and manufactured food products. T3.4—Characterisation of the manufactured food products. T3.5—Identification of acceptability markers.	Pulses suitable for consumer expectations and farmers' needs in France and minimally processed pulse‐based IFPs with preserved nutritional quality and functional properties. Innovative recipes with the IFPs, suitable for both home use and industrial production. Development of savoury flans and biscuits based on pulse flours. Understanding of the effect of pulse flour incorporation on aroma release and consumer sensory perception. Acceptability markers related to sensory, physicochemical and textural properties.
WP4	T4.1—Building a suitable LCA model. T4.2—Life cycle assessment of the territorialized legume chain. T4.3—Integration of analyses and modelling.	LCA model for agricultural pulse production to be applied for various types of technical itineraries. LCA inventory data sets for pulse production and transformation technologically and geographically representative. Scenarios of territorialized pulse‐based food chains in Bourgogne Franche‐Comté (BFC) and corresponding results of local environmental impacts, ecosystem services systems and socio‐economic performances of agricultural systems and local food‐chains.
WP5	T5.1—Assessment of the costs and benefits of cooking pulses in a kitchen lab. T5.2—Evaluation of the costs and benefits of consuming pulse‐based products in a restaurant setting. T5.3—Comparison of consumer behaviours regarding innovative legume products: from home to collective restaurants.	Understand consumer motivations, barriers, and levers for using legume‐based ingredients in real‐life contexts. Identify perceived costs/benefits influencing acceptance. Assess perceived sensory, acceptability, practical costs and benefits of cooking legumes in a controlled kitchen lab. Identify drivers, barriers and waste behaviours, evaluate attitudes and acceptance of legume‐based products in collective catering. Compare home vs. restaurant behaviours to identify context effects on acceptance and consumption. Provide recommendations to enhance legume adoption.
WP6	T6.1—Mapping discourses on pulse‐centred innovative solutions. T6.2—Co‐construction and testing of messages. T6.3—Impact assessment of communication strategies.	Comprehensive understanding of how innovation and sustainability narratives are framed in public and professional discourse related to pulses. Development of communication strategies co‐created with diverse consumer groups and refined by professional designers. Experimental evaluation of message clarity, comprehension and engagement levels through online surveys and focus groups. Identification of effective communication levers to foster consumer acceptance and behavioural change towards pulse‐based food innovations. Integration of findings to provide actionable recommendations for policymakers, industry stakeholders, and communication professionals, ensuring alignment with insights and prototypes developed in other WPs.

## 2 Work Package 1—Identification of the Process‐Related Barriers That Limit the Consumption of Pulses

Driven by the CSGA (Centre des Sciences du Goût et de l'Alimentation), WP1 will provide all the partners with insights into consumers' mental representations, attitudes, habits and expectations towards processing of pulses. The objectives are (i) to identify the cognitive and attitudinal barriers that explain the low adoption of minimally processed pulses; (ii) to characterise the motivations for cooking and preparing pulse‐based dishes at home among those who consume pulses; (iii) to understand the differences in consumer representation between minimally processed and ultra‐processed pulse‐based foods; and (iv) to identify solutions that could meet consumer expectations regarding pulses to pave the way for WP3.

Food choices are complex behaviors that depend on multiple interacting factors, including consumer personalities, food properties, contextual influences and broader social and structural environments (Ogden 2011; Shepherd 2001). WP1 addresses the key consumer‐related barriers to pulse consumption. The acceptance and choices of specific foods are strongly influenced by familiarity with those foods, which is gained through previous experience. Moreover, attitudes, traits and expectations also influence acceptability and choice, and the reasons driving food choice are not easily accessible. Asking consumers to explain their choices or expectations regarding food is particularly challenging, often leading to memory retrieval errors or social desirability bias (King and Bruner 2000; Fisher and Katz 2000). Consequently, if we want to base innovations on consumer expectations, we cannot simply question them about the properties they would like to find in new products, and we will also need to use indirect and/or implicit methodologies that measure cognitive processes that may not be accessible through direct self‐reports or introspection. Indirect approaches assess behaviors and physiological responses, whereas implicit tests capture automatic cognitive processes that are difficult for individuals to consciously report (Monnery‐Patris and Chambaron 2020; Zsoldos et al. 2022; Melendrez‐Ruiz, Buatois, et al. 2019; Melendrez‐Ruiz, Chambaron, et al. 2019).

### 2.1 Identification of Mental Representations and Attitudes Towards Pulses With Different Levels and Types of Processing

Authors will conduct a survey (aiming to capture responses from roughly 150 people, with profiles representative of the French population according to the quota method), and indirect tasks will examine the differences in consumers' minds between minimally processed and ultra‐processed pulse‐based foods (e.g., chickpeas cooked in water and falafels). Indirect tasks are experimental approaches in which participants do not have to explicitly report what they think or how they usually behave. Participants rather have to carry out specific tasks, such as composing a menu (Melendrez‐Ruiz, Buatois, et al. 2019; Melendrez‐Ruiz, Chambaron, et al. 2019) or associating items. This type of approach allows researchers to avoid some biases that frequently occur with direct questioning, such as social desirability bias. Additionally, we assess respondents' nonconscious attitudes towards food processing by an implicit test that measures the strengths of the associations between the level of processing (i.e., the concept) and various chosen attributes (e.g., sensory acceptance, health and convenience of cooking).

### 2.2 Observation of Current Consumers' Culinary Practices Concerning Home‐Preparation of Pulses

This study will be based on an observational approach, very classical in ethnographic field. As such, the idea will not be to control any parameter but to observe people with various profiles in their usual environment. This task determines whether a lack of knowledge, resources (cooking tools and skills), or time availability makes it difficult to prepare recipes containing pulses at home. (i) Participants who regularly cook pulses will be observed in their own kitchens. They will be observed and videotaped while cooking pulses as they usually do. Then, the participants will be asked questions related to their cooking practices and habits with pulses (e.g., know‐how, soaking, cooking time but also who taught them to cook pulses, when and how). The perceptions of different methods of cooking pulses, as well as their possible tips to save time and make the preparation easier, will be assessed. This will allow us to obtain feedback from the participants about the cooking conditions, events and cultural backgrounds that facilitated their habit of cooking pulses. (ii) To explore barriers to pulse cooking, innovative focus groups should be conducted in a cooking workshop. Traditional focus groups involve having people interact in an open discussion, with the aim of expressing ideas related to a particular topic. When new products are validated, the discussion is based on product tasting. Here, we propose a discussion organised around the collective task of cooking pulses. The participant profiles will vary, with people of different socioeconomic statuses and different habits and attitudes towards pulses, to encourage debate and possibly include different opinions or attitudes.

### 2.3 Identifying Consumer‐Centred Solutions and Cocreation for WP3

The key findings from the focus groups will inform the design of a quantitative survey to further explore consumer beliefs, preferences and attitudes towards pulse consumption and preparation. To achieve these insights, cocreation with consumers will be carried out on the basis of the model of ‘ideathons’ or participatory research. This process will bring together individuals from diverse backgrounds, skills and expertise to diagnose predefined problems, identify promising opportunities, and generate innovative, viable solutions. The goal is to collaboratively design solutions that address barriers to pulse consumption and contribute to the development of more structured, consumer‐oriented pulse value chains.

## 3 Work Package 2—Obstacles and Drivers for the Structuring of Pulse Value Chains

The cultivation of pulses has several environmental and nutritional benefits. Their presence in rotations allows the use of fertilisers, especially chemical fertilisers, to be reduced. The nutritional properties of pulses are recognised both for animal and human consumption (Schneider and Huyghe 2015). However, the cultivation of these crops remains limited due to obstacles related to the supply and demand of pulses. Many obstacles to the diversification of pulses have been identified in the literature: the lack of investment by crop breeders in minor species, due to the limited market; the small number of studies on these species by technical institutes and researchers; and the significant investment and risk‐taking that are perceived by farmers in modifying their cropping systems (lack of references and information). Additionally, the logistical organisation of agricultural cooperatives and traders that is not always adapted to the management of minority crops (Magrini et al. 2013), as their organisations are closely linked to their marketing strategies (Fares et al. 2012). The difficulties in developing niche channels such as pulses can be explained by the existence of sociotechnical lock‐in. The origin of a lock‐in is most often multifactorial, social and technical and is linked to the path dependence of most innovations (Magrini et al. 2016; Stassart and Jamar 2012; Vanloqueren and Baret 2009). For example, the establishment of contracts in the context of minor crops has several objectives: greater uncertainty in production (qualitative dimension, yields), a lack of knowledge and technical references on these crops, and impacts in terms of the reconfiguration of rotation practices and, more generally, the redesign of the farmers' cropping systems (Cholez et al. 2017; Cholez 2019; Cholez and Magrini 2023).

Moreover, pulses grown for human consumption represent limited outlets. Despite being more lucrative than livestock feed, the human pulse food market is very limited in size compared with that of animal feed. Only chickpeas, lentils and a small proportion of soybeans are used for human consumption. In addition, the market is dominated by imported products rather than products ‘made in France’. It appears crucial to structure the chains or microchains of these crops (Daniel and Margetic 2021). At present, these crops are still confined to niche production. The development of new markets, particularly for human food, requires the development of communication with consumers but must also result in innovations in terms of products and processes. The outlet for industrial ingredients for food companies represents, for example, an outlet that is twice as important as for whole seeds (mainly lentils, beans and chickpeas) (Baret and Vanloqueren 2011). Thus, it is essential to coordinate the efforts of many actors and establish a mode of organisation and governance to regulate the supply of products on the market in relation to demand at the risk of price collapse. The transition requires an understanding of the lock‐in of the sociotechnical regimes of current food systems (Baret and Vanloqueren 2011; Meynard et al. 2018), the construction of alternatives, and a political framework for these alternatives to be built and to support the transition (Jensen et al. 2012). The analysis of pulse chains in France (Cholez 2019) highlights the crucial role of establishing a collective organisational structure like a cooperative or an interprofessional organisation, for example. This governance structure is able to coordinate the actions and learning of all actors within the sector and ensure its sustainability.

We have three objectives in WP2.

### 3.1 Identify the Main Actors, Territories and Characteristics of Pulse Production and Processing in France and Bourgogne Franche‐Comté (BFC)

The first step is to conduct an overview of the main pulse producing regions in France, the volumes of pulse production and the main actors involved in France and in BFC in the field of pulses for human consumption (e.g., cooperatives, producer groups, processing companies, associations, local authorities and collective catering, and retailers). We identify the different quality and value‐adding labels that characterise raw and processed pulse‐based products and their associated volumes (AB, or other ‘agroecological’ labels). The project should help to better define, through exchanges between WPs, what a legume‐based product can be according to the proportion of legume‐based ingredients and their position in the list of ingredients, given that there is not necessarily a consensus definition (Salord et al. 2024). Based on surveys of stakeholders involved in the production and processing of pulses, we identify and evaluate the different forms in which pulses are found (e.g., bulk, raw but packaged, canned (heat‐sterilised), or in the form of functional ingredients) and the distribution channels (mass distribution, specialised distribution and other industrial enterprises in the case of B2B distribution).

The research will be conducted as follows: (1) by analysing existing documentation (reports and studies), databases (such as General Agricultural Survey) and professional press and (2) by using semi‐structured interviews with expert resource stakeholders of the pulse sector (interprofessional bodies, technical centres, researchers, institutions, economic actors such as cooperatives and distributors).

### 3.2 Determine the Main Locks (Technical, Economic, Sociological) Related to the Production and Processing of Pulses and Identify Innovations (Technical, Organisational and Marketing) and Individual and Collective Initiatives for the Production and Processing of Pulses Introduced

In addition to the identification of pulses produced in BFC, this work consists of a more detailed analysis of the value chains of a few pulses that will be chosen as emblematic of local production and used for human consumption. On the basis of semi‐structured interviews with regional actors that are involved in different links of the value chain, the objectives are to (1) determine how value is shared between stakeholders throughout the supply chain in conventional supply chains and those benefiting from a label; (2) identify the technical, organisational and economic obstacles to the development of these pulses; (3) identify the incentive systems (contracts^1^ and economic incentives) that have been put in place; and (4) identify innovative initiatives of territorialized chains of pulses.

The results obtained will contribute to the identification of the pulses that are most interesting for processing and marketing and will therefore contribute to the determination of the products tested in WP3. Once the products have been developed, it will be possible to conduct a series of interviews with manufacturers and retailers to test the interest of the products developed.

### 3.3 Collect Data to Contribute to Life Cycle Assessment (WP4)

The target is to contribute to the establishment of credible scenarios in the territorial context of BFC. This task consists of carrying out quantitative data collection with the identified actors in the value chains in BFC: farmers, cooperatives, storage organisations and processors. The purpose will be to build hypotheses on material and energy flows into the BFC territory in relation to the production and processing of pulses for human consumption. The objective of WP4 is to assess the environmental benefits of developing pulse chains in BFC by implementing prospective simulations of agronomic, socioeconomic and environmental data at the territory scale. The objective is to propose scenarios in WP4 that reflect the real behaviours of the actors. The surveys conducted as part of WP2 should enable the identification of different types of organisations among existing legume production and processing stakeholders in the BFC territory. Value chain scenarios are established from socioeconomic data.

## 4 Work Package 3—Development and Characterisation of Minimally Processed Reformulated Foods That Are Compatible With Consumer Expectations

This work package (WP3) is a cornerstone of PULSE FICTION and focuses on the following:

– Selecting and characterising ingredients and locally grown pulses with no a priori restrictions (i.e., considering all possible grain pulses options for human consumption), on the basis of insights from consumer barriers (WP1) and production and processing challenges (WP2).
– Developing recipes using intermediate food products (IFPs), which are suitable for both home cooking and industrial scale, and analyzing their physico‐chemical, sensory and textural properties.
– Identifying key acceptability markers on the basis of consumer preferences, sensory perceptions and product characteristics.

### 4.1 Selection and Processing of Ingredients

A selection of local pulses will be processed to create IFPs, such as flour, aquafaba and precooked raw or crushed grain pulses, intended for integration into various recipes. The selection criteria will remain open, ensuring that only pulses meeting the WP1 and WP2 requirements are considered. Approximately 10 IFPs will be developed. The selection of IFPs will be conducted by an internal expert panel, considering trade‐off dimensions such as identified needs from WP1 and WP2, selected pulses (i.e., the type of IFP most appropriate for these pulses), low processing, nutritional and functional properties, and innovative approaches.

### 4.2 Characterisation of the Selected Intermediate Food Products

The selected IFPs will undergo compositional analysis, including the following:

– Nutrient profiling (e.g., protein, total sugars, starch, fibre content, lipid composition, minerals and anti‐nutritional compounds).
– Functional property assessment (e.g., water and oil absorption, emulsifying activity, stability, viscoelastic properties and swelling).

These properties will be evaluated using standard analytical methods to determine their suitability for culinary applications.

### 4.3 Integration of IFPs in Culinary Recipes and Manufactured Food Products

#### 4.3.1 Culinary Recipes

The IFPs will be incorporated into innovative recipes designed by a chef, considering their functional properties (WP3.2) and consumer insights (WP1). These recipes will:

– Be designed without preconceptions, ensuring flexibility in formulation. This principle can be advanced using a culinary design method proposed by the chef (Grégoire Maille, personal communication, 25 March 2024) which includes the following phases: definition of specifications, creative research taking into consideration the selected IFPs, recipe development and transmission.
– Be partially or fully able to substitute for traditional protein sources.

Approximately 40 recipes will be developed, ensuring that they are both practical for home cooking and scalable for industrial production.

#### 4.3.2 Manufactured Food Products

Three different flours with three different levels of pulse incorporation will be used to create savoury flans and biscuits. Each food will be compared against a corresponding reference (without pulses) to analyse the nutritional, sensory and textural differences.

### 4.4 Characterisation of Manufactured Food

Compositional (as described in Section 2) and textural analyses will be used to characterise all the foods designed in Section 3. These will be adapted according to the characteristics of the foods under consideration. These analyses will be completed by sensory tests. The panel will be composed of individuals between 18 and 65 years old, with a good balance between men and women, following an omnivorous or flexitarian diet and with no allergies to pulses. The sensory tests in this section will not include additional information about the benefits of pulses (e.g., nutritional and environmental benefits) given to consumers to evaluate its influence on sensory perception. For savoury flans and biscuits, the different levels of incorporation of pulse flours will allow the comparison of their impacts on different properties of these products. The simultaneous use of dynamic sensory and instrumental measurements will make it possible to establish a link between the release of aromatic compounds in the mouth and their perception over time using temporal sensory evaluations coupled with PTRMS (Proton Transfer Reaction Mass Spectrometry). These analyses can be used to characterise these foods to optimally identify acceptability markers.

### 4.5 Identification of Acceptability Markers

The set of data obtained in Section 4 is related to the set of product characteristics obtained in Section 2. In particular, a multivariate approach will be used to reveal the relationships between sensory perception, physicochemical composition and textural data of the products before and during their consumption. All the data will be compared with the acceptability data obtained in WP5 to identify acceptability markers.

## 5 Work Package 4—Multicriteria Analysis of Territorialized Food Chains of Pulse‐Based Products

The objective of this WP is to assess the environmental benefits of developing pulse chains in BFC by implementing prospective simulations of agronomic, socioeconomic and environmental data at the territory scale. We will use Integrated Assessment Modelling (IAM) to simulate spatialized environmental, economic and social impacts at the local level (Wohlfahrt et al. 2019; Misslin et al. 2022). For this, we will first perform Life Cycle Assessment (LCA) of whole pulse chains. LCA is a method used to calculate a large set of impact indicators (e.g., Green house gas (GHG) emissions, water eutrophication, acidification of soil and water and ecotoxicity) that is related to a product while accounting for the impacts from the whole value chain of this product from agricultural production to food consumption (Sala et al. 2017). Then, LCA models will be coupled with IAM to simulate scenarios of combined changes in agricultural activities, biomass processing and recycling.

The work will be closely linked to WP2, as the collected data and results of WP2 will be used as input data for LCA and to build prospective scenarios for IAM.

Three tasks have been defined.

### 5.1 Building a Suitable LCA Model

Accounting for pulse‐modified rotations for the delivery of human and livestock nutrition in the LCA model is still a challenge. We propose an appropriate LCA approach, framed in the BFC context, to assess the environmental sustainability of agri‐food system transition effectively.

### 5.2 LCA of the Territorialized Pulse Chain

Quantitative data collection will be carried out with identified actors in the value chains of Bourgogne Franche‐Comté (BFC) farmers, cooperatives, storage organisations and processors. The purpose will be to build hypotheses on the material and energy flows into the BFC territory in relation to the production of pulses for human consumption. Value chain scenarios will be established from socioeconomic data collected in WP2 and modelled by LCA for the calculation of environmental impacts. We will also evaluate the environmental consequences of substituting food items at the dietary level, with consumption hypotheses deduced from the results of WP1.

### 5.3 Integrated Analyses and Modelling

The opportunities provided by the development of pulses in a territory must be cautiously balanced with other possible scenarios and potential risks, considering the complex social and ecological dynamics of agricultural systems (Ewert et al. 2009). The potential increase in the production and human consumption of pulses in BFC will be integrated and modelled through the MAELIA Platform (INRAE, Paris, France) (http://maelia‐platform.inra.fr/), a valuable tool to support ideas and discussions about the profound and necessary restructuring of agroecosystems to address social–ecological challenges at the local level. Currently, the platform allows us to represent, at fine spatial and temporal resolutions, the dynamics of and interactions between agricultural activities (e.g., choosing crop rotation and conducting different cropping systems within each production system), the functions of biomass processing and recycling plants, the hydrology of different water resources and water resource management, and climate change. This platform has been used to assess the environmental and socioeconomic sustainability and resilience of scenarios regarding exchanges of pulses between cereal farmers and livestock farmers (Catarino et al. 2021), recycling of rural and urban organic wastes (Misslin et al. 2022), and spatial upscaling of agroecological systems (Tribouillois, Constantin, Casal, et al. 2022; Tribouillois, Constantin, Murgue, et al. 2022). The work performed will help extend the functionalities of this platform to address issues related to pulse‐based food chains and consumption and assess, through LCA, exported environmental impacts, that is, impacts linked to the production of synthetic and organic inputs of agricultural systems and food processing plants.

## 6 Work Package 5—Consumer‐Perceived Costs and Benefits of Different Options for the Consumer and How This Impacts the Final Acceptability

Understanding consumer motivation, as well as the key drivers and limits to incorporating new pulse ingredients or products into meal preparation, is essential. To ensure realistic insights and minimise potential biases, it is crucial to collect real‐life data on preparation and consumption behaviours. The use of experimental platforms allows us to observe consumers in a real‐life setting while maintaining the rigour of experimental science. In WP5, we will evaluate the consumer‐perceived costs (e.g., sensory, financial, effort‐related, preparation time and nutritional) and benefits associated with cooking pulse‐based ingredients and recipes. We will also examine how these factors influence the final acceptability of pulse‐based products in two distinct contexts: a home kitchen and a collective restaurant.

### 6.1 Costs and Benefits to Cook Pulses in a Kitchen Lab

To assess the perceived costs and benefits perceived by consumers during the preparation and consumption of pulse‐based products, two steps are proposed:

– Evaluation of sensory properties, financial cost, preparation time and preparation difficulty perceived by consumers in charge of preparing meals at home during the preparation of the different recipes developed in WP3. Their preparation behaviors will be recorded in the *SayFood Kitchen Lab* using video analysis, followed by post preparation interviews to obtain qualitative insights.
– Assessment of the impact of these costs and benefits on consumer perception and appreciation. For this purpose, participants will be invited to consume and evaluate pulse‐developed products in a meal context and in an ecological situation. These measurements will be completed by evaluating the levels of perceived satiation and satiety through a questionnaire on food intake during the next meal. In addition, the glycaemic index will be measured under in vivo or in vitro conditions (to be determined).

### 6.2 Costs and Benefits of Consuming Pulse‐Based Products in a Restaurant Setting

The selection of products is evaluated in the context of ecological consumption. The objective is to analyse consumer behaviours and perceptions regarding these products when they are integrated into a real food offering. This will be conducted in the experimental restaurant of L'Institut Agro Dijon, which is equipped to allow the observation of consumers at lunchtime in real life. First, participants' food choices will be collected to assess to what extent and for what reasons the products developed in this project are selected over other available choices. Second, the reactions of those participants who chose the products developed in WP3 will be collected through an immersive or participatory observation method. The observations will allow us to collect information on the possible discussions between the consumers about the products, as well as the way they handle the products, observe them, season them, cut them up, separate them and eventually divert part of them to the edge of the plate. If products are not fully consumed, the wasted part will be weighed. Finally, a questionnaire will be administered to further explore explicit attitudes and perceptions towards these new pulse‐based products. With this approach, the collected data on eating behaviours will help identify and better understand the barriers and drivers associated with pulse‐based products to promote healthier and more sustainable diets.

### 6.3 Consumer Behaviours of Innovative Pulse Products: From Home to Collective Restaurants

This task aims to compare the consumption of innovative pulse‐based products in two contexts: at home and in collective catering. The goal is to analyse the differences in adoption and acceptance depending on the consumption environment. The main variables characterising the consumption environment could include physical context (meal setting, availability, preparation mode), social context (presence of others, norms, communication), psychological aspects (autonomy, attitudes, familiarity) and institutional factors (policies, cost, staff practices). These dimensions differ strongly between home consumption and collective catering, shaping consumers' acceptance of innovative pulse‐based products.

## 7 Work Package 6—Development of Messages in Support of Consumer Appropriation of Pulse‐Centred Innovative Solutions

This work package (WP6) aims to design and assess effective communication strategies that enhance consumer engagement with pulse‐centred innovative food products. The approach is structured around three key objectives:

(i) Mapping and analysing existing discourses that promote pulses and pulse‐based innovations, including messages from the food industry, startups and public institutions.

(ii) Co‐constructing communication messages with consumers, ensuring their alignment with consumer expectations and concerns.

(iii) Evaluating the impact of these messages on consumer adoption through experimental exposure and behavioral assessment.

### 7.1 Mapping Discourses on Pulse‐Centred Innovative Solutions

A systematic discourse analysis will be conducted to identify the key messaging trends and persuasive strategies used by various stakeholders, including the food industry, startups and public institutions (e.g., Ministries of Health, Agriculture and Ecology). The document base will include institutional, corporate and media communications published between 2017 and 2025, covering campaigns, websites, reports and social media posts related to pulse‐based innovations. A mixed text‐mining approach combining keyword co‐occurrence, topic modelling (LDA) and qualitative discourse analysis will be used to map dominant narratives and framing strategies across stakeholder groups. The expected outcome is a map of communication landscapes, allowing for a deeper understanding of the dominant narratives shaping consumer perceptions of pulses and their roles in sustainable food systems.

### 7.2 Co‐Construction and Testing of Messages

A participatory approach will be employed to co‐construct visual and textual communication elements with a diverse panel of food citizens, ensuring representation across age, dietary habits, geographical areas and socio‐professional categories. Focus groups will facilitate the development of messages that resonate with consumer expectations. These messages will then be refined by professional designers and tested through an online survey to assess the clarity, comprehension and engagement levels. The professional designers involved will be experts in visual communication and food design, with experience in translating scientific and behavioural insights into accessible, consumer‐oriented materials. Their role will be to ensure visual coherence, aesthetic appeal and alignment with the communication objectives defined during the co‐construction phase. Two distinct messaging strategies will be selected: one emphasising innovation and the other sustainability.

### 7.3 Impact Assessment of Communication Strategies

To evaluate the effectiveness of the developed messages, an experimental study will be conducted. The participants will be divided into three groups: (i) a control group with no exposure to the messages and (ii) two experimental groups, each exposed to one of the selected communication strategies. Following exposure, participants will engage in a practical cooking task using a pulse‐based recipe developed by a chef. Their perceptions, attitudes and willingness to integrate pulse‐based innovations into their diets will be assessed through a structured questionnaire.

This work package provides valuable insights into the role of strategic communication in shaping consumer engagement with sustainable food innovations. The pulse‐centred solutions addressed here will be defined and developed based on the technological, nutritional and sensory outcomes generated in the preceding work packages. By integrating discourse analysis, participatory design and experimental validation, WP6 contributes to identifying the most effective strategies for fostering the adoption of these pulse‐centred food solutions, offering valuable implications for policymakers, industry stakeholders and communication professionals.

## 8 Innovations

### 8.1 Adopt a Consumer‐ and Farmer‐Centred Approach

The design of innovative products should consider the expectations of consumers and the factors that could constitute obstacles to the adoption of the new products. Indeed, expert opinions and scientific knowledge do not guarantee that consumers will react positively to and adopt a new product. It has become quite common to involve consumers in the validation of food innovations by asking them to give their opinions in the form of sensory evaluations or willingness to pay. It is less common to integrate a deep understanding of consumer practices and habits into the innovation process (Kylkilahti et al. 2022). In regard to pulse‐based product development, it seems necessary to move from firm‐driven innovations to a more integrative approach in which the expectations of consumers and producers are considered. Indeed, different representations have been reported between pulse‐chain stakeholders, who mainly associate pulses with their protein content and culinary preparation, and consumers, who rather evoke the taste of pulses and barely distinguish them from starchy foods (Melendrez‐Ruiz et al. 2020). Moreover, the environmental impacts of food in consumers' minds only partially overlap with those considered in the LCA (Schiano and Drake 2021). The PULSE FICTION project takes a novel approach by placing both consumer insights (WP1) and farmer constraints (WP2) at the core of innovation. Furthermore, in WP6, we will codevelop communication strategies with consumers to ensure their engagement and promote pulse‐based solutions effectively.

### 8.2 Direct Measurement Biases Should Be Avoided When Consumers' Representations and Attitudes Towards Pulse Processing Are Addressed

Real‐life choices are often guided by nonconscious rather than conscious mechanisms, so it is particularly difficult for consumers to verbalise their expectations and representations of food products. This has been illustrated by an ideation exercise about new plant‐based products that was conducted during a focus group (Varela et al. 2022), in which participants struggled to come up with suggestions. Even when participants are asked to give their opinions about an existing food product, their answers can be biased. For example, participants who guess the objective of the study can be tempted to provide answers that they believe will be viewed favourably by others rather than giving their honest opinion. This social desirability bias might be particularly strong in regard to studying people's opinions about pulses, whose health and environmental benefits are highly publicised by the scientific community and the government. In a previous study, participants reported positive attitudes towards minimally processed or unprocessed pulses despite consuming very few of these products (Melendrez‐Ruiz et al. 2022). Any innovations based on participants' declarations could thus be misleading and result in inadequate development paths. Relying solely on self‐reported preferences may lead to misguided product development. To counteract this, WP1 will incorporate advanced research methods from psychology and anthropology to capture implicit attitudes and cognitive biases towards minimally processed pulse‐based foods.

### 8.3 Enhancing Consumer Acceptance by Addressing Off‐Flavour and Identifying Acceptability Markers

Pulse products are rejected by some consumers because of their unappreciated sensory properties. These products are often described as ‘green,’ ‘grassy,’ ‘beany,’ ‘fatty’ and/or ‘bitter’ (Karolkowski et al. 2021, 2023). The compounds responsible for these off‐flavours are formed through various pathways, including lipid oxidation, ethanol fermentation and the Maillard reaction (and Strecker degradation). The generation of off‐flavour compounds in plants is strongly affected by the species, cultivar, geographical location, climate conditions and farming and harvest practices (Leonard et al. 2022). WP3 of PULSE FICTION addresses the challenge of obtaining slightly processed food whose sensory properties are acceptable to consumers. For this purpose, we will play on different levers, such as the origin and variety of crops, the process to obtain IFPs, the recipes created by the chef and the invention of new ways to consume pulses.

### 8.4 The Development of Methodologies for Performing Integrated Assessments and Modelling of the Increasing Consumption of Pulse‐Based Products From a Territorial Perspective

Another goal of our project is that the products and recipes implemented will have to meet sustainability requirements. A LCA method will be adapted to the specific details of pulse production, and at the same time, the environmental consequences of substituted food items at the dietary level will be evaluated. LCA data will be integrated with geographical and socioeconomic data to create prospective scenarios. Two complementary tools will be used to model pulse production: at the farm step and at the whole food chain scale (WP4).

This territorial approach will help policy‐makers and industry stakeholders better understand the systemic impacts of increasing pulse consumption.

### 8.5 This Approach Addresses the Challenge of Combining Realistic Environments and Rigorous Data Collection in Consumer Science

Consumer food choices and acceptance are strongly shaped by context; yet, most studies are conducted in artificial laboratory settings (Best et al. 2018; Dacremont and Sester 2019). The neutrality of the premises, the presence of the experimenter and the absence of social cues, which are usually considered in the presence of other eaters, modify the behaviours and responses of participants. Sensory perceptions, social dynamics and habitual behaviours all influence how a food product is perceived and adopted in daily life. Setting up study conditions allowing participants to be immersed in a realistic environment, which does not affect perceptual, attentional, or decision‐making processes while guaranteeing satisfactory data collection conditions, is a real methodological challenge. In WP5, we propose new approaches to study consumer acceptance of the pulse‐based recipes developed by the chef. We combine methodologies from consumer science and participatory research and use them in innovative settings that place consumers in a real‐life context of dish preparation or consumption: a ‘kitchen laboratory’ and an ‘experimental restaurant’. This approach combines consumer science and participatory research to ensure accurate, ecologically valid insights into pulse adoption.

## 9 Impact

PULSE FICTION is based on a consumer‐ and farmer‐oriented approach to understand the needs and expectations of minimally processed pulse‐based products to develop products that meet these needs. Its other purpose is to adopt a sustainability‐oriented approach, focusing on the environmental, social and economic sustainability of the entire pulse value chain, from production to consumption. A key differentiator of PULSE FICTION is its holistic sustainability focus, which integrates environmental, social and economic dimensions across the entire pulse value chain—from production to consumption. The project's impact extends beyond individual stakeholders, benefiting consumers, farmers, industry and society as a whole.

### 9.1 Impact on Consumers

PULSE FICTION will break new ground in understanding the barriers to the consumption of minimally processed pulse‐based products. Its original approach provides an understanding of consumer expectations before products are designed, in opposition to the usual strategy, which consists of assessing the sensory defects of products and convincing consumers to accept them by persuasion or implementing masking strategies. PULSE FICTION provides new evidence on how consumers consider the processing of food products and a global vision of how to improve the acceptability of pulses by French consumers, resulting in the development of products that are much more in line with consumer expectations.

Another benefit of the PULSE FICTION project is the proposal of recipes with appealing sensory properties that are easily usable by consumers at home to avoid the most common sources of rejection. Thus, the cook is brought into the kitchen for coaching in the preparation of pulse‐based foods in the consumer's home. This contrasts with most of the work already done on pulses, where consumer studies are performed away from home. For the first time, we define a multifactorial acceptability index that considers physicochemical constraints, the determinants of adoption by consumers (e.g., sensory, practicability and digestibility) and LCA. This concept, if it appears relevant, could be used more generally in other studies.

### 9.2 Impact on the Pulse Chain Stakeholder

PULSE FICTION fosters a dynamic, multistakeholder ecosystem, connecting industrial partners, farmers, scientists and consumers through innovation from ‘forks, fields and factories’. The consumption of all types of pulses will increase in the coming years. Production and processing must follow. Therefore, there is great potential for the industry in this area, provided that the products are well accepted by the consumer. Our recipes aim at increasing pulse acceptance and adoption by French consumers, which in turn will provide more incentives to French farmers to grow them by ensuring outlets for pulses through their more frequent and regular consumption. The recipes based on pulses will be adaptable to an industrial scale. Consumer acceptance testing and a consumer behaviour study of PULSE FICTION will ensure that the products formulated with pulses will be not only acceptable but also attractive to consumers and thus indirectly increase the competitiveness of the producing industry. Thus, our project outcomes will provide industry with guidelines for the development of more sustainable food products with nutritional and health benefits. By bridging the gap between consumer acceptance and industrial feasibility, PULSE FICTION lays the foundation for a thriving, home‐grown pulse sector.

### 9.3 Societal Impact

PULSE FICTION, by offering acceptable and easy recipes based on pulses, should contribute to the introduction of more plant proteins into the French diet at the expense of animal proteins and thus improve consumer health. Plant proteins are superior to animal proteins in preventing chronic diseases, as shown by the EPIC‐Oxford study of 58 056 European people (EPIC‐Oxford 2023). Although plant proteins generally contain less lysine and methionine than do those of animal origin, this deficiency can be compensated for by dietary variations. The consumption of sulphur amino acids in greater quantities in animal proteins is associated with a greater risk of cardiometabolic diseases, such as those caused by elevated blood cholesterol, glucose, uric acid, insulin and glycated haemoglobin levels, and certain cancers (Dong et al. 2018). In particular, colorectal cancer is one of the four cancers responsible for half of health care costs, and its incidence is constantly increasing every year (Klimeck et al. 2023; Jafari et al. 2024). Thus, the outcomes of PULSE FICTION could contribute to reducing public health costs. Notably, the total cost, according to health insurance records, for colorectal cancer cures was 1700 million euros in 2017. In parallel, a reduction in meat consumption would be beneficial for the environment and the French socioeconomic landscape.

## 10 Conclusion: PULSE FICTION as a Model for Future Sustainable Food Innovation

By integrating consumer insights, sustainable farming and scalable industry solutions, PULSE FICTION is at the forefront of food system transformation. Its impacts extend beyond product development, establishing the groundwork for healthier diets, a more resilient agricultural sector and enhanced environmental sustainability. The consumer‐centric approach adopted in PULSE FICTION is key to its success: by understanding the cognitive barriers to pulse consumption, the project will produce recommendations of innovations that are likely to be accepted by consumers and that will form the starting point for a reorganisation of the sector. Indeed, any innovative product is doomed to failure if consumers do not adopt it. Our approach will make it easier to identify the types of innovation that can be adopted by consumers, thus limiting the risks for players in the pulse sector.

This initiative has the potential to redefine the future of pulses in France, demonstrating that sustainability and consumers are not mutually exclusive but mutually reinforcing.

The interdisciplinary and participatory framework developed in PULSE FICTION—combining consumer science, psychology, food processing, sustainability assessment and stakeholder engagement—offers a robust model that may be adapted and replicated to other categories of under‐consumed but health‐promoting foods.

Specifically, this approach may benefit the promotion of foods such as whole grains, nuts, seeds or certain vegetables, which often face similar barriers to those observed for pulses: low familiarity, poor culinary integration, negative sensory representations, or a lack of consumer‐friendly formats. These food groups, despite their recognised nutritional value, often remain marginal in current dietary patterns due to perceptual and behavioural obstacles rather than objective limitations.

By applying a similar logic—grounding innovation in consumer expectations, minimising unnecessary processing and enhancing culinary usability—it is possible to broaden the impact of sustainable dietary transitions across multiple food categories. We believe that the methodology piloted in PULSE FICTION can serve as a model for future initiatives aimed at reshaping eating habits in favour of health and environmental resilience.

## 11 PULSE FICTION Team

WP1—Pr Gaelle Arvisenet (L'Institut Agro—CSGA, leader); Dr. Stephanie Chambaron (INRAE—CSGA, co‐leader); Dr. Emmanuelle Ricaud‐Oneto (L'Institut Agro—CSGA) and Dr. Solène Leprince (INRAE—CSGA).

WP2—Pr Corinne Tanguy (L'Institut Agro—CESAER, leader); Abdoul Diallo (L'Institut Agro—CESAER); Dr. Monia Saidi (L'Institut Agro—CESAER); Dr. Hélène Gérard‐Simonin (L'Institut Agro—PAM); Dr. Mélanie Favrot (L'Institut Agro—CESAER); and Marie Dubot (Terres UNIVIA).

WP3—Dr. Christian Salles (INRAE‐ CSGA, PI and leader); Zaira Hernandez‐Casiano (INRAE—CSGA); Grégoire Maille (Restau'Co); Dr. Caroline Peltier (INRAE—CSGA), Isabelle Andriot (INRAE—CSGA); Géraldine Lucchi (INRAE—CSGA); Karine Gourrat (INRAE—CSGA); Olivier Euvrard (Bouvard Alina Industrie); Cécile Lebrun (Bouvard Alina Industrie); Pr Camille Loupiac (L'institut Agro—PAM); Dr. Hélène Labouré (L'institut Agro—CSGA) and Dr. Hélène Labouré (L'institut Agro—CSGA) and Dr. Hélène Gérard‐Simonin (L'Institut Agro—PAM).

WP4—Pr Hélène Gérard‐Simonin (L'Institut Agro—PAM, leader); Pr Camille Loupiac (L'Institut Agro—PAM); and Dr. Olivier Theron (L'Institut Agro—LEA).

WP5—Pr Anne Saint‐Eve (AgroParisTech—Sayfood, leader); Dr. Valérie Guénard‐Lampron (AgroParisTech—Sayfood); Dr. Lucy Espinosa‐Brisset (AgroParisTech—Sayfood); David Forest (INRAE—Sayfood); Dr. Carole Tournier (INRAE—CSGA); Pr Gaelle Arvisenet (L'Institut Agro—CSGA); Pr Catherine Dacremont (L'Institut Agro—CSGA); and Dr. Emmanuelle Ricaud‐Oneto (L'Institut Agro—CSGA).

WP6—Pr Clémentine Hugol‐Gential (Université Bourgogne Europe—CIMEOS, leader); Dr Estera Badau (Université Bourgogne Europe—CIMEOS); Dr Cyril Masselot (Université Bourgogne Europe—CIMEOS); Dr Fabien Bonnet (Université Bourgogne Europe—CIMEOS); and Dr Aude Chauviat (Université de Bourgogne Europe—CIMEOS).

## Author Contributions

**Gaëlle Arvisenet:** writing – review and editing, writing – original draft, supervision, project administration, investigation, conceptualisation. **Stéphanie Chambaron:** writing – review and editing, writing – original draft, supervision, project administration, investigation, conceptualisation. **Zaira Hernandez‐Casiano:** writing – review and editing, writing – original draft, supervision, project administration, investigation. **Hélène Gerard‐Simonin:** writing – review and editing, writing – original draft, supervision, investigation, conceptualisation. **Corinne Tanguy:** writing – review and editing, writing – original draft, supervision, investigation, conceptualisation. **Clémentine Hugol‐Gential:** writing – review and editing, writing – original draft, supervision, investigation, conceptualisation. **Anne Saint‐Eve:** writing – review and editing, writing – original draft, supervision, investigation, conceptualisation. **Christian Salles:** writing – review and editing, writing – original draft, supervision, project administration, investigation, conceptualisation.

## Funding

This work was granted state aid managed by the French National Research Agency under France 2030 programme with the reference 23‐PLEG‐0004.

## Conflicts of Interest

The authors declare no conflicts of interest.

## Data Availability

The data that support the findings of this study are available from the corresponding authors upon reasonable request.

## Endnotes

^1^
Contracts could have two effects: sharing risks, giving economic premium, supporting learnings through specific investment in human capital (Magrini et al. 2023, 2025).
